# Current concept on assessment and management of anterior cruciate ligament injury in skeletally immature athletes

**DOI:** 10.1002/jeo2.70498

**Published:** 2025-11-16

**Authors:** Alberto Grassi, Kyle Borque, Claudio Rossi, Bruna Cascone, Francesco Della Villa, Stefano Zaffagnini

**Affiliations:** ^1^ Dipartimento di Scienze Biomediche e Neuromotorie (DIBINEM) University of Bologna Bologna Italy; ^2^ II Clinica Ortopedica e Traumatologica IRCCS Istituto Ortopedico Rizzoli Bologna Italy; ^3^ Department of Orthopaedic Surgery Houston Methodist Hospital Houston Texas USA

**Keywords:** anterior cruciate ligament, arthroscopy, knee, paediatric, skeletally immature

## Abstract

The incidence of anterior cruciate ligament (ACL) injuries in paediatric and skeletally immature patients is rising, proportionally to the worldwide spread of sport participation. However, differently from adults, injuries in young patients pose serious challenges regarding their management, especially when surgery is required. To date, controversies remain regarding the indications, timing of surgery and techniques for ACL reconstruction. This current concept provides a deep and detailed theoretical background of ACL injury and management in patients with open physis. Moreover, a practical algorithm of treatment is provided based on authors' experience and research and current evidence‐based medicine (EBM). To optimise management and reduce the risks of either conservative or surgical treatment, a deep knowledge of the specific concepts related to paediatric patients is required.

**Level of Evidence:**

Level V.

**Abbreviations**
ACL: anterior cruciate ligament
AE: all‐epiphyseal
EBM: evidence‐based medicine
IKDC: International Knee Documentation Committee
IOC: International Olympic Committee
ITB: iliotibial band
LEAP: lateral extra‐articular procedures
LET: lateral extrarticular tenodesis
MAT: movement analysis test
MTPF: menisco‐tibia‐popliteo‐fibular
PCL: posterior cruciate ligament
PLUTO: paediatric ACL: understanding treatment options
ROM: range of motion
STEP: sagittal tibial epi‐physis

## INTRODUCTION

The incidence of anterior cruciate ligament (ACL) injuries in paediatric and skeletally immature patients is rising, proportionally to the worldwide spread of sport participation [18, 47]. However, differently from adults, injuries in young patients pose serious challenges regarding their management, especially when surgery is required [50]. In fact, if the nonoperative treatment is burdened by a high incidence of subsequent meniscal injuries [48, 49, 83], the surgical reconstruction could be responsible of iatrogenic growth abnormalities and limb deformities [24].

To date, despite the International Olympic Committee (IOC) developed a consensus paper on the management of paediatric ACL injuries [6, 71], controversies remain regarding the indications, timing of surgery and techniques for ACL reconstruction. Thus, the aim of this study is to provide a framework for the assessment and management of skeletally immature patients with ACL injury based on authors experience and current EBM.

## THE BURDEN OF ACL INJURY IN SKELETALLY IMMATURE PATIENTS

**Figure 1:**
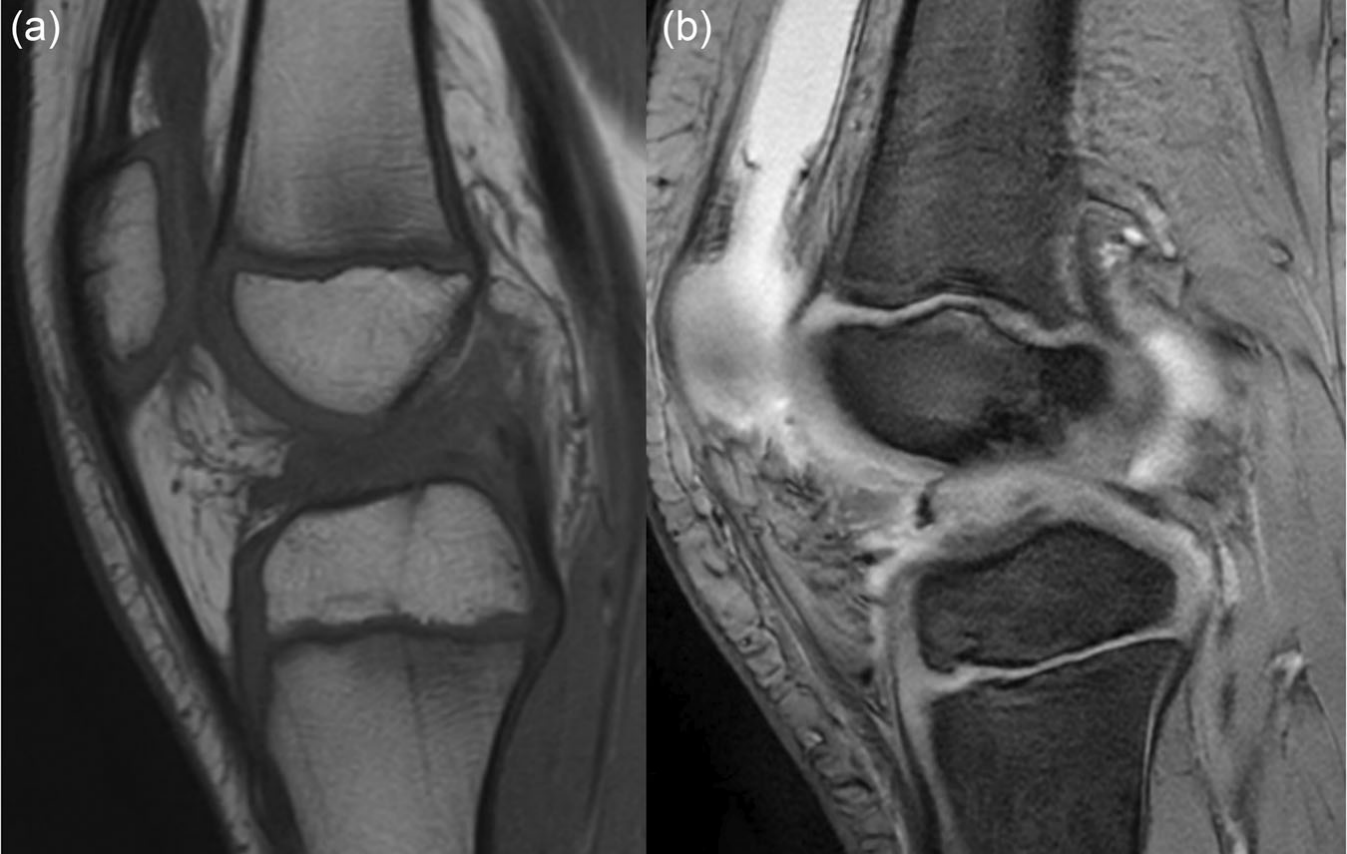
Nine‐year‐old male patient with complete anterior cruciate ligament (ACL) injury after direct knee injury during soccer practice (a); 6‐year‐old female with complete ACL injury occurred while skiing (b).

ACL tears in young skeletally immature patients account for less than 5% of all ACL injuries [74], rarely before the age of nine and more commonly between the age of 12 and 14. The number of paediatric ACL tears have been increased over the years at an annual rate of 2.3% in the United States [11], either due to the growing popularity of high‐risk sports in children and adolescents [6, 86] (Figure 1) and the physicians' improved clinical and diagnostic skills [80].

Historically, these injuries were managed conservatively due to concerns about potential damage to the growth plates during surgical intervention [57]. The traditional approach discouraged sports participation and advised patients to wait until skeletal maturity before considering surgical options. However, this strategy has proven to be largely unsuccessful and has two primary drawbacks. First, the psychological impact on young patients is considerable [46]. Being unable to participate in sports or physical activities they enjoy can lead to feelings of isolation, frustration, lower self‐confidence, depression and loss of social networks [46, 80]. For active children, this can severely affect their quality of life and overall well‐being. Second, and perhaps more critically, is the issue of knee instability. An unstable knee increases the risk of subsequent meniscal injuries. Studies indicate that the incidence of meniscal tears in paediatric patients with unstable knees is significantly higher, with some reports suggesting rates as high as 60%–70% [64, 85]. These meniscal injuries can be particularly problematic because they often necessitate partial or total meniscectomy when lesions are not promptly treated. The long‐term consequences of meniscectomy are well‐documented: research shows that patients who undergo meniscectomy are at a higher risk of developing osteoarthritis later in life [67]. This increased risk of osteoarthritis means that an ACL injury in childhood can have lifelong repercussions, fundamentally altering the patient's future joint health and quality of life. Thus, ACL injuries in paediatric patients are not just a temporary setback but can lead to a lifetime of complications if not managed appropriately. Considering early surgical intervention represents a crucial step in improving long‐term outcomes for these young patients, allowing them to maintain an active lifestyle and reducing the risk of future joint problems. However, before undertaking the treatment of this particular population of patients, the clinician should be aware of the particular process of skeletal growth, be familiar with the bone age assessment and handle the specific physeal sparing techniques for ACL reconstruction.

## CONSIDERATIONS ON SKELETAL GROWTH

The knowledge of the basis of overall skeletal and knee growth [51] is important when managing skeletally immature patients with ACL rupture. In particular, when ACL reconstruction is indicated, different surgical techniques could be needed based on the skeletal age and remaining growth, to minimise the risk of iatrogenic injuries. In the following pages, the general principles of overall knee growth, growth predictions and skeletal age assessment are presented; however, it is important to note that this information provides only a partial reflection of the growth phenomena and should be interpreted critically [51].

### Overall growth

Generally, after a rapid growth up to the 5th year of life, there is a marked deceleration in growth between 5 and 10 years of age, with standing height increasing approximately 5.5 cm per year. After the age of 10 years, the growth patterns of boys and girls diverge: on average, girls experience the onset of puberty at the age of 11, boys at the age of 13. There are wide individual variations in onset, tempo and duration of puberty, which could influence the ultimate patient's height [51].

**Figure 2:**
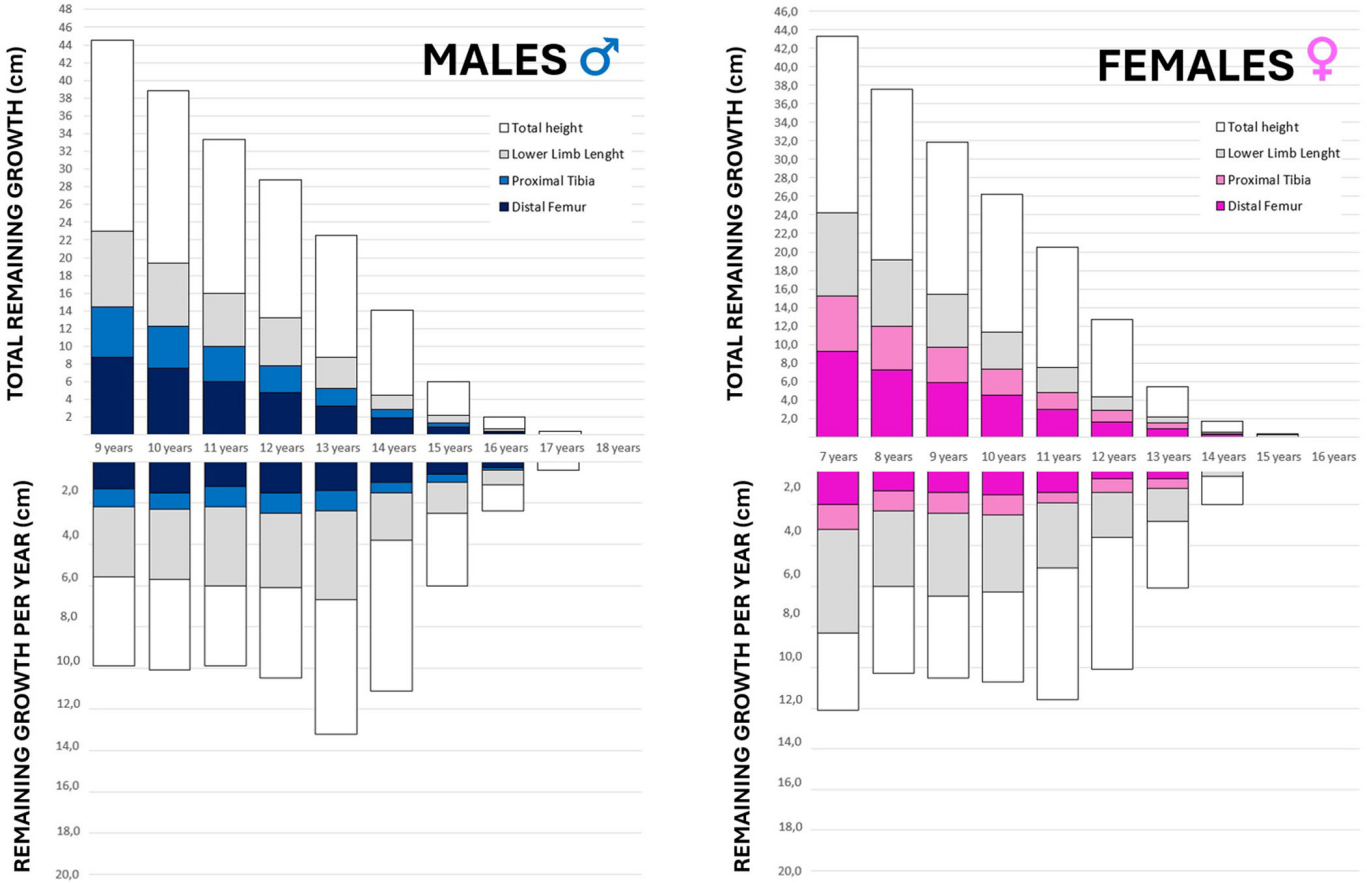
Bar charts of remaining growth at a given age and per year, for males and female patients, based on the data from Kelly and Dimeglio [51].

During puberty, standing height increases by approximately 1 cm per month. At the onset of puberty, boys have remaining standing height approximately 22.5 cm (9.5 cm in leg length), while girls have remaining standing height of approximately 20.5 cm (8.5 cm in leg length). The first phase of the pubertal growth spurt corresponds to the acceleration in the velocity of growth: this phase lasts 2 years from approximately 13–15 years of bone age in boys and from 11 to 13 years of bone age in girls. The gain in standing height for boys during this phase is approximately 16.5 cm (8 cm in leg length) while the gain in standing height for girls is approximately 14.5 cm (7 cm in leg length). The second phase of the pubertal growth spurt is a period of deceleration in the rate of growth, which lasts 2.5 years from 15 to 17.5 years of bone age in boys and from 13 to 15.5 years of bone age in girls. During this phase, boys and girls gain approximately 6 cm in standing height, with only 1.5 cm gain in leg length [51]. At the bone age of 16 year for males and 14 years for females, lower limbs growth comes virtually to a standstill [62] (Figure 2).

### Knee growth

The cycle of growth in the lower limb and knee are very predictable: there is a rapid growth during the first 5 years of age, followed by a slower growth from 5 years of age to the onset of the puberty, when a growth spurt occurs [51]. The femur grows more than the tibia but with a constant relationship between each other which is set at an early stage. The distal femoral growth plates grow approximately 1.0 cm per year. During puberty, this rate of growth increases to approximately 1.2 cm [51]. The tibial growth profile is almost identical to the femoral one. Overall, the remaining growth of the knee joint (distal femur and proximal tibia) at the beginning of puberty is nearly 3 cm. According to Menelaus et al. [62], skeletal maturity of the distal femur and proximal tibia is reached at the age of 16 in boys and at the age of 14 in girls (Figure 2).

### Growth predictions

The prediction of the remaining growth at a given time point is important to balance the risks and benefits of various treatment options. There are methods that predict height, lower limb growth, knee development or tibial and femoral length.

The Menelaus arithmetic method described in 1966 [62] is the simplest method to determine how much growth remains around a patient's knee. It is based on the principles that skeletal growth ends at the age of 16 in boys and at the age of 14 in girls, and that the distal femur provides 9 mm of growth per year, while the proximal tibia provides 6 mm per year. Thus, the remaining growth is thus calculated multiplying the value of 15 (6 + 9 mm) for the remaining years of growth to skeletal maturity. Despite the Menelaus method does not consider the fact that growth rate is not constant, it is simple and rather reliable.

The Anderson‐Green growth remaining method [5] has been developed plotting the skeletal age (determined with the Greulich and Pyle atlas using left hand radiograph) against growth remaining of the distal femur and the proximal tibia. Each Anderson‐Green graph has five lines based on mean values and −2, −1, +1, +2 Standard Deviations. These charts are helpful but not considered as completely accurate today, therefore the Moseley method [70] has been developed to mathematically reformatting the Anderson‐Green data into straight‐line graphs representing limb lengths. However, this method requires to plot multiple data points over time to obtain the prediction.

Dimeglio et al. described skeletal growth from childhood to skeletal maturity [51]. The authors formatted their data on growth remaining into a graphical format that facilitates the prediction of growth remaining from the physes of the knee; moreover, they evaluated the subischial length, the sitting height and overall height. Their charts directly relate bone age to growth remaining and took into account the different rate of growth along the years, thus making their data a good tool to stratify patients preoperatively according to the remaining knee growth.

Finally, Paley and his colleagues developed several methods [73] to estimate the overall height, the length of femur, of the tibia and of the whole lower limb, based on multipliers obtained from limb length databases. According to a given chronological age, the growth remaining of the different segments could be simply calculated using specific multipliers.

In the current practice of the authors, the Dimeglio charts [51] are used to assess the preoperative remaining growth of the knee and estimate the surgical risk, while the Paley methods are used to provide an estimation of height and lower limb length at maturity in order to monitor the patients until skeletal maturity.

## SKELETAL AGE ASSESSMENT

**Figure 3:**
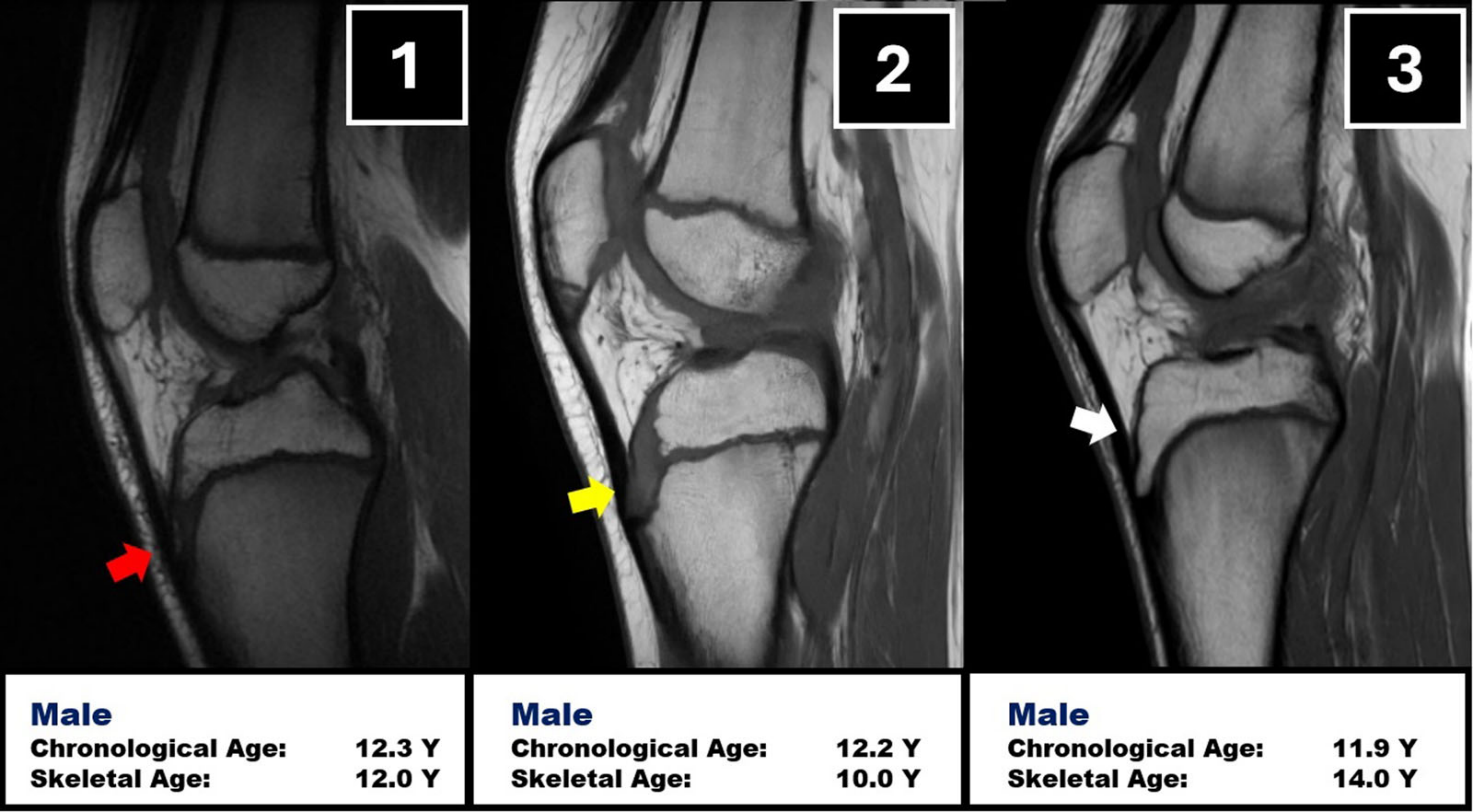
A series of three skeletally immature patients with a chronological age of 12 years that shows different status of tibial tubercle ossification and thus with different skeletal age (yellow arrow: absence of ossification nucleus; red arrow: presence of ossification nucleus; white arrow: complete ossification of tibial tubercle).

Most of the previous considerations on skeletal growth are not based on chronological age but on skeletal age. Thus, it is mandatory to properly assess skeletal age in adolescent patients, since it does not always overlaps with chronological age. To this regard, Grassi et al. [42] compared the chronological age with the skeletal age of 79 skeletally immature patients with ACL injury. It was reported that in more than 25% cases there was a mismatch of at least 1 year between the two ages. Moreover, among the kids which should have reached skeletal maturity according to chronological age, 15% of them had still open physes and more than 1 year of remaining growth, thus requiring a specific ACL reconstruction technique [42] (Figure 3).

Regarding age assessment, the Tanner scale based on the development of secondary sexual characteristics (pubic hair, testicular growth and breast development) has been used historically to identify physiological age. However, Tanner scale has been demonstrated to have a poor correlation with skeletal age [12] and a that orthopaedic surgeons are not reliable and accurate in its assessment [82]. Thus, the Tanner scale is not included in the Algorithm of treatment, despite most authors used to dichotomise skeletally immature patients undergoing ACL reconstruction as Tanner I–II (no or sparse pubic hair, no enlargement of breast and areola, no growth of the penis) when prepubescent, and as Tanner III–V (darker pubic hair, enlargement of breast and areola, growth of penis) when pubescent adolescents.

Regarding bone age assessment, the most widely utilised reference is the atlas of Greulich and Pyle, which is based on a single left‐hand‐wrist radiograph [10]. Despite its widespread use, it has the limitation of not considering that skeletal age assessed from wrist radiographs could vary from that assessed with knee radiographs [1] and thus this approach could not be appropriate when treating knee pathologies. Moreover, this method requires the execution and interpretation of an additional radiological imaging not directly related to the involved joint. To overcome these limitations, Pennock et al. [75] developed an atlas of knee MRIs spanning the paediatric and adolescent years, that enabled accurate assessment of skeletal age and that had a similar reliability of the Greulich and Pyle atlas. Further studies [63, 78] created a shorthand approach to determine skeletal maturity based on the previous atlas, with an excellent inter‐ and intrarater reliability, even superior to the hand atlas. These shorthand atlases are based on the interpretation of several MRI features, with whom the orthopaedic surgeon should be more familiar:

### Patellar ossification

When incomplete more than 50%, it identifies ages <10 years in males and <8 years in females. The superior tip of the patella is the last to ossify and can be still present in older kids.

### Tibial tubercle extension of the epiphysis

It is assessed in the sagittal T‐1 slices, and is present when the ossification of the proximal tibial epiphysis is extending downward towards the tibial tubercle; however, the ossification centre of the tibial tuberosity itself is not present yet, thus the overall development could be too incomplete to drill osseous tunnels through the epiphysis.

### Presence of tibial tubercle apophyseal centre

It is assessed in the sagittal T‐1 slices, and it is identified when a discrete ossification nucleus inferiorly with respect to the tibial physis can be identified. It indicates that tibial tubercle is starting to develop, and that the maturation of the proximal epiphysis is almost complete; thus, it could be suitable to drill a sovra‐physeal tunnel drilling without damaging the growth cartilage.

### Presence of tibial tubercle ‘crack’

it is assessed in the sagittal T‐1 slices, and is identified as a thin hypointense line separating the tibial epiphysis and the tibial tubercle apophysis. It indicates that the two structures are near to the complete fusion between with each other.

### ‘Oreo’ sign

it is assessed on sagittal T‐1 slices, especially at the level of the femoral condyles. It is defined as a laminated appearance of the subchondral epiphyseal cartilage, usually as black‐grey‐black layers. When the subchondral cartilage presents as a single black layer, the ‘Oreo’ sign is considered to be disappeared.

### Tibial and femoral growth cartilage status

it is evaluated on coronal T‐1 slices, where the course of growth cartilage can be assessed from the peripheral portion to the centre of the bone. According to its status, the cartilage could be labelled as ‘completely visible’, ‘partially closed’ (usually in its central portion) and ‘completely close’. When still completely visible, the growth cartilage could have a ‘thick’ appearance (>1.5 mm thickness) or a ‘thin’ appearance (<1.5 mm thickness) based on the development status [30]. Usually, tibial cartilage becomes thinner and closes earlier respect to the femoral one; thus, the combination of their status could aid in the assessment of skeletal age.

## SAGITTAL TIBIAL EPI‐PHYSIS (STEP) SHORTHAND FOR MRI BONE AGE ASSESSMENT

**Figure 4:**
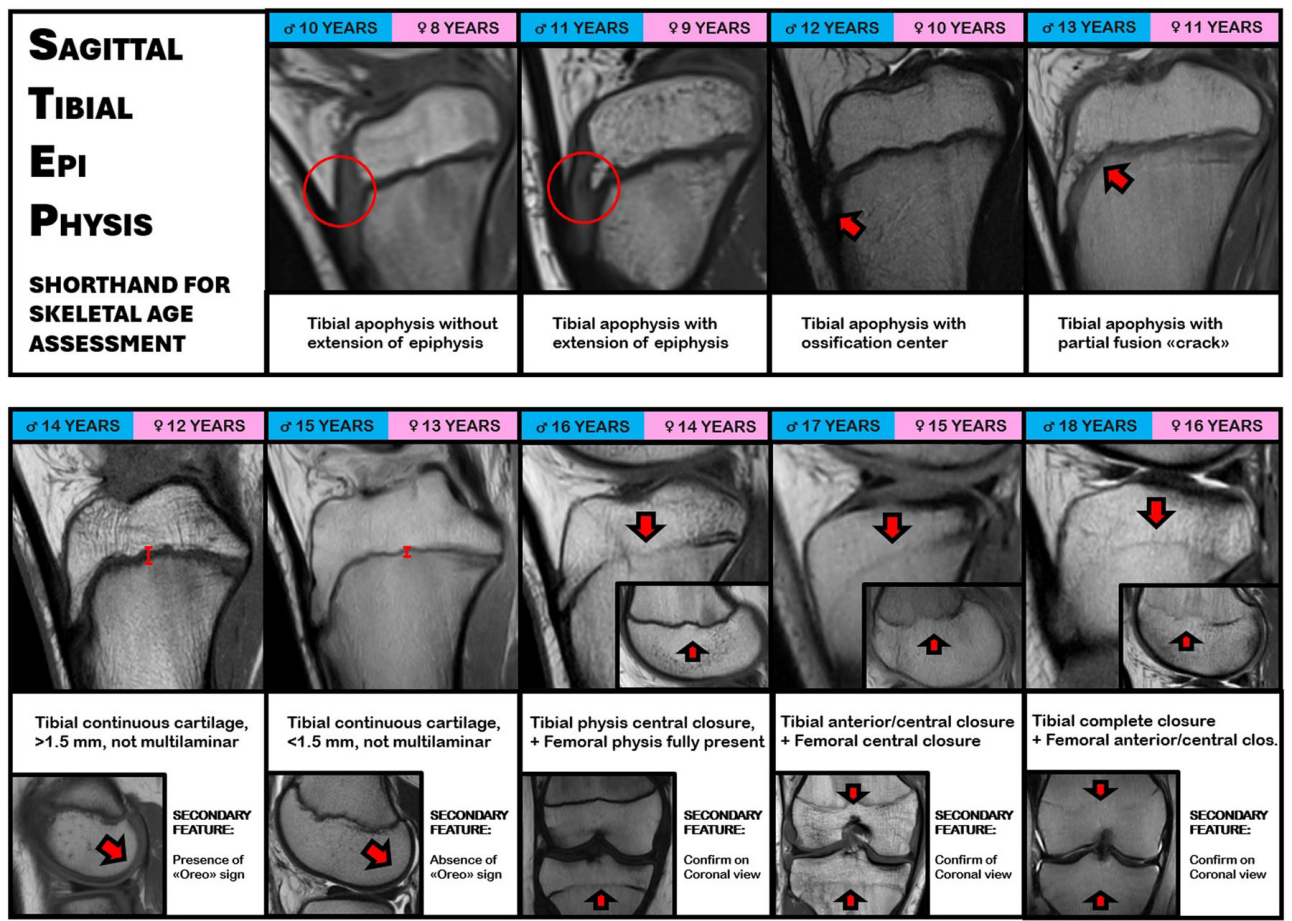
Sagittal tibial epi‐physis (STEP) shorthand for skeletal age assessment based on knee magnetic resonance imaging (MRI).

Using these characteristics, the authors created a shorthand atlas based on knee MRI called STEP, which can determine skeletal age based on the assessment of the tibial epiphysis in sagittal MRI slices (Grassi 2025, personal communication) (Figure 4). By evaluating 130 MRIs from 97 skeletally immature patients with ACL injury, the authors reported a good correlation between each age milestone with the median age of patients in that specific age group (Table 1). Moreover, the evaluation focused only on tibial ossification status simplify the assessment of skeletal age, which is based on a ‘continuum’ of phases clearly identifiable through MRI.

**Table 1:** Median age for each age milestone according to the sagittal tibia epi‐physis (STEP) shorthand.

	Males (*n* = 103)			Females (*n* = 27)		
Magnetic resonance imaging feature	Age ref. (years)	Median age (years)	Patient n°	Age ref. (years)	Median age (years)	Patient n°
Tibial apophysis without extension of epiphysis	<11	**9.4**	12	<9	**‐**	0
Tibial apophysis with extension of epiphysis	11	11.2	6	9	**‐**	0
Tibial apophysis with ossification centre	12	12.3	6	10	**‐**	0
Tibial apophysis with partial fusion ‘crack’	13	13.1	6	11	**‐**	0
Tibial continuous cartilage, >1.5 mm, not multilaminar	14	14.1	20	12	11.7	1
Tibial continuous cartilage, <1.5 mm, not multilaminar	15	15.1	18	13	12.8	5
Tibial physis central closure, Femoral physis fully present	16	15.9	13	14	14.1	7
Tibial physis anterior and central closure, Femoral physis central closure	17	16.7	17	15	14.8	7
Tibial physis complete closure, Femoral physis anterior and central closure	18	17.9	5	16	15.8	7

*Note*: Moreover, according to the combinations of these features, the authors identify three age groups (Table 2):

**Table 2:** Characteristics of each age milestone according to the sagittal tibia epi‐physis (STEP) shorthand.

		**Bone age (years)**	
	**Features**	**Females**	**Males**
Prepubescents	Tibial apophysis without extension of epiphysis	8	10
	Tibial apophysis with extension of epiphysis	9	11
Young adolescents	Tibial apophysis with the presence of distal ossification centre	10	12
	Tibial apophysis with partial fusion to the epiphysis and with ‘crack’ line	11	13
	Tibial apophysis completely closed and tibial growth cartilage completely present, with ‘thick’ thickness >1.5 mm and without multilaminar appearance. As a secondary feature, the ‘Oreo’ sign is still visible	12	14
	Tibial apophysis completely closed and tibial growth cartilage completely present, with ‘thin’ thickness <1.5 mm and without multilaminar appearance. As a secondary feature, the ‘Oreo’ sign is not visible	13	15
Old adolescents	Tibial growth cartilage is closing only in the central portion, with femoral growth cartilage still completely visible. As a secondary feature, the status is confirmed on coronal slices	14	16
	Tibial growth cartilage is closed in the anterior and central portion, while femoral growth cartilage is closing only in its central portion. As a secondary feature, the status is confirmed on coronal slices	15	17
	Tibial growth cartilage is (almost) completely closed, while femoral growth cartilage closed in its anterior and central portions. As a secondary feature, the status is confirmed on coronal slices	16	18

*Prepubescents (males < 12 years, females < 10 years):* the main MRI criteria to characterise this group of patients is the *absence of tibial tubercle apophyseal centre*; moreover, both tibial and femoral physis are completely visible and with a ‘thick’ appearance. These patients still have 1 or more years before the puberty onset and the remining growth at the level of the knee exceed 7.5 cm, especially on the distal femur (5 cm). At least 5 years remain until knee skeletal maturity.

*Young adolescents (males 12–15 years, females 10–13 years):* the main MRI criteria to characterise this group of patients is the *presence of tibial tubercle apophyseal centre* and both *tibial and femoral physis completely visible, either* ‘thick’ or ‘thin’. These patients are close or into the first phase of pubertal growth spurt, where the remaining growth at the level of the knee is between 7.5 and 1 cm, with a fair amount on the distal femoral side (3–0.5 mm). Four to one year remains until knee skeletal maturity.

*Old adolescents (males 16–18 years, females 14–16 years):* the main MRI criteria to characterise this group is the presence of *tibial physis partially or completely closed*. These patients finished the puberty phase or are into its late phase, where the remining growth at the level of the knee is less than 1 cm. The time to complete knee skeletal maturity is less than 1 year.

## INITIAL MANAGEMENT AND INDICATIONS FOR SURGERY

When the basis of skeletal growth and bone age assessment are familiar, a better and more standardised management of ACL injury can be delivered. Usually, the ACL tear is a consequence of a noncontact injury involving cutting, pivoting or rapid deceleration. In an acute setting, intense pain and hemarthrosis can be present impeding a precise examination and the execution of laxity tests. It is important not to miss the initial diagnosis, since it is possible that patients referred for a dislocated meniscus bucket handle tear may have an underlying, previously undiagnosed ACL injury [80].

When a proper diagnosis of acute rupture has been performed by means of clinical examination, X‐ray and high‐quality MRI, a treatment plan is necessary. Its goal are: (1) to restore a stable, well‐functioning knee that enables a healthy, active lifestyle across the lifespan; (2) to reduce the impact of existing or the risk of further meniscal or chondral pathology, degenerative joint changes and the need for future surgical intervention; (3) to minimise the risk of growth arrest and femur and tibia deformity [6, 80].

The two possible treatment options that can help the child to achieve these goals are high‐quality rehabilitation alone or ACL reconstruction. According to the IOC guidelines, surgery should be reserved for the cases with (1) associated meniscus or cartilage lesions; (2) recurrent, symptomatic giving way; (3) unacceptable restriction in participation to sports or recreational activities. In all other cases, a conservative approach including bracing and a home‐based 3–6 months rehabilitation program are encouraged; this could be a short‐term option to delay surgery until skeletal maturity or even a permanent treatment if no further lesion progression occurs [6].

However, despite the conservative treatment is considered safe, as it does not have the complications of surgery, it has several drawbacks. First, return to Level I sports (sports with frequent pivoting and contact, for example, soccer, handball, basketball) [44] should be considered with caution [80] due to the high risk of recurrent sprain, especially in the case of high rotatory laxity. Therefore, changing physical activity to level 2 sports (mostly individual sports with less frequent pivoting than level I sports, for example, racket sports, alpine skiing, snowboarding, gymnastics and aerobics) could be considered an acceptable option for children with lower ambitions but can have negative psychological effects in young competitive athletes and their families. The use of braces during competitive activity could be problematic as well, due to possible compliance issues or reduction of sport performance during dexterity sports like soccer or basket.

### The role of meniscal lesions

**Figure 5:**
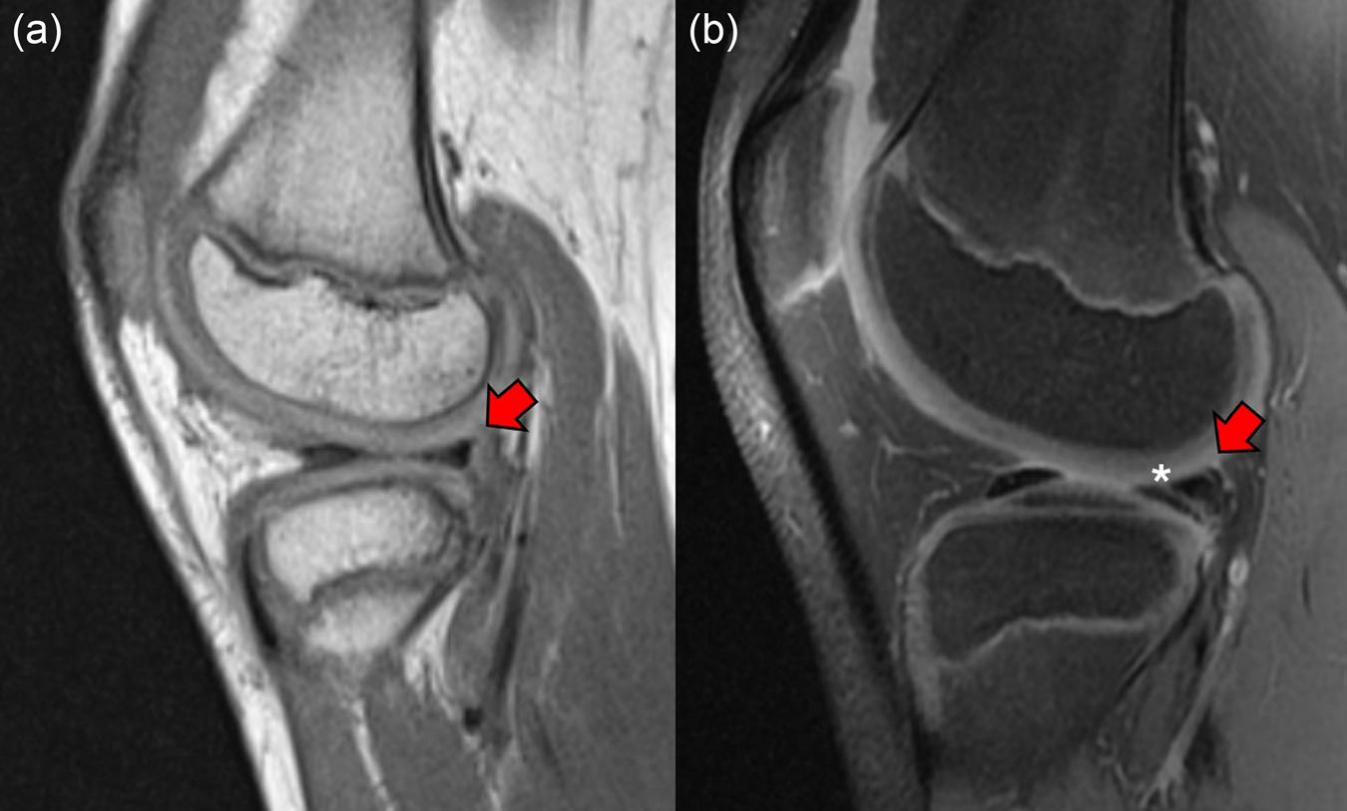
Complete anterior cruciate ligament (ACL) rupture in a 9.5‐year‐old male with intact lateral meniscus (red arrow) managed conservatively (a). After 18 months, a secondary longitudinal tear of the posterior horn (white asterisk) is seen, together with the onset of locking symptoms (b).

**Figure 6:**
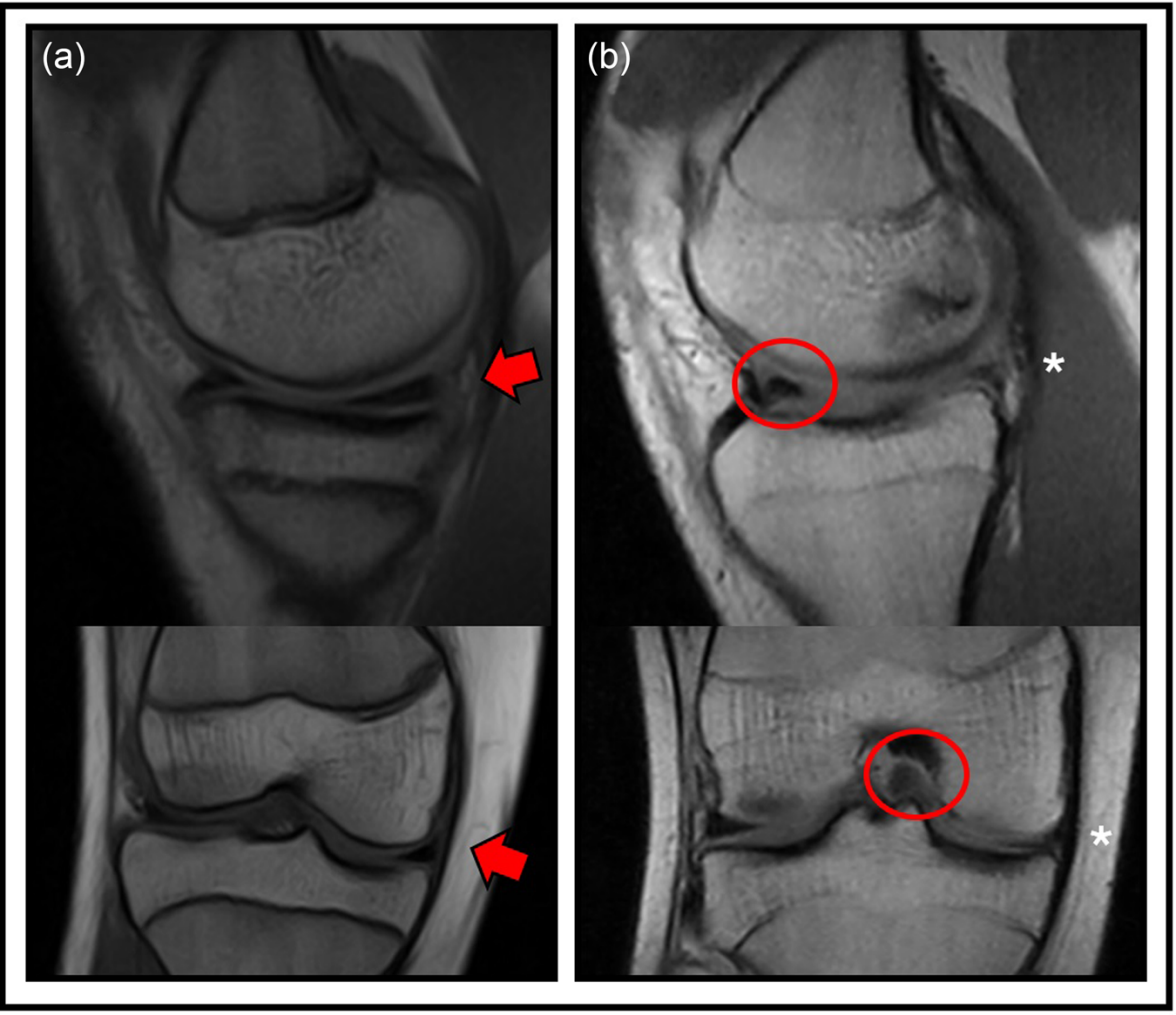
Complete anterior cruciate ligament (ACL) rupture in a 14.5‐year‐old male with intact medial meniscus (red arrow) managed conservatively (a). After 15 months, a secondary bucket handle tear of the medial meniscus displaced in the notch (red circle) is seen, together with sudden knee locking and pain (b).

Another concern of conservative treatment, apart from the high risk of cross‐treatment to surgery, is the possible development of secondary meniscal tears or the worsening of pre‐existing tears (Figure 5). Grassi et al. [37] performed a retrospective assessment of 151 consecutive ACL reconstruction in patients with less than 16 years of age and a mean age of 14.8 years. Those who had surgery within 3 months from injury had less meniscal lesions compared to those waiting more than 6 months (36% vs. 55%) and less severe lesions compared to those waiting more than 12 months (7% vs. 20%) (Figure 6). Moreover, a higher chance of meniscal repairability was present in those with early surgery (92% vs. 46%). This represents a relevant finding, since a higher chance of having residual pain during activity 6 years after ACL reconstruction was noted in patients with lateral meniscectomy compared to those with intact meniscus (56% vs. 26%). Thus, all effort should be applied to reduce the meniscal injury rate and their possible impact on long‐term clinical outcomes [37].

**Figure 7:**
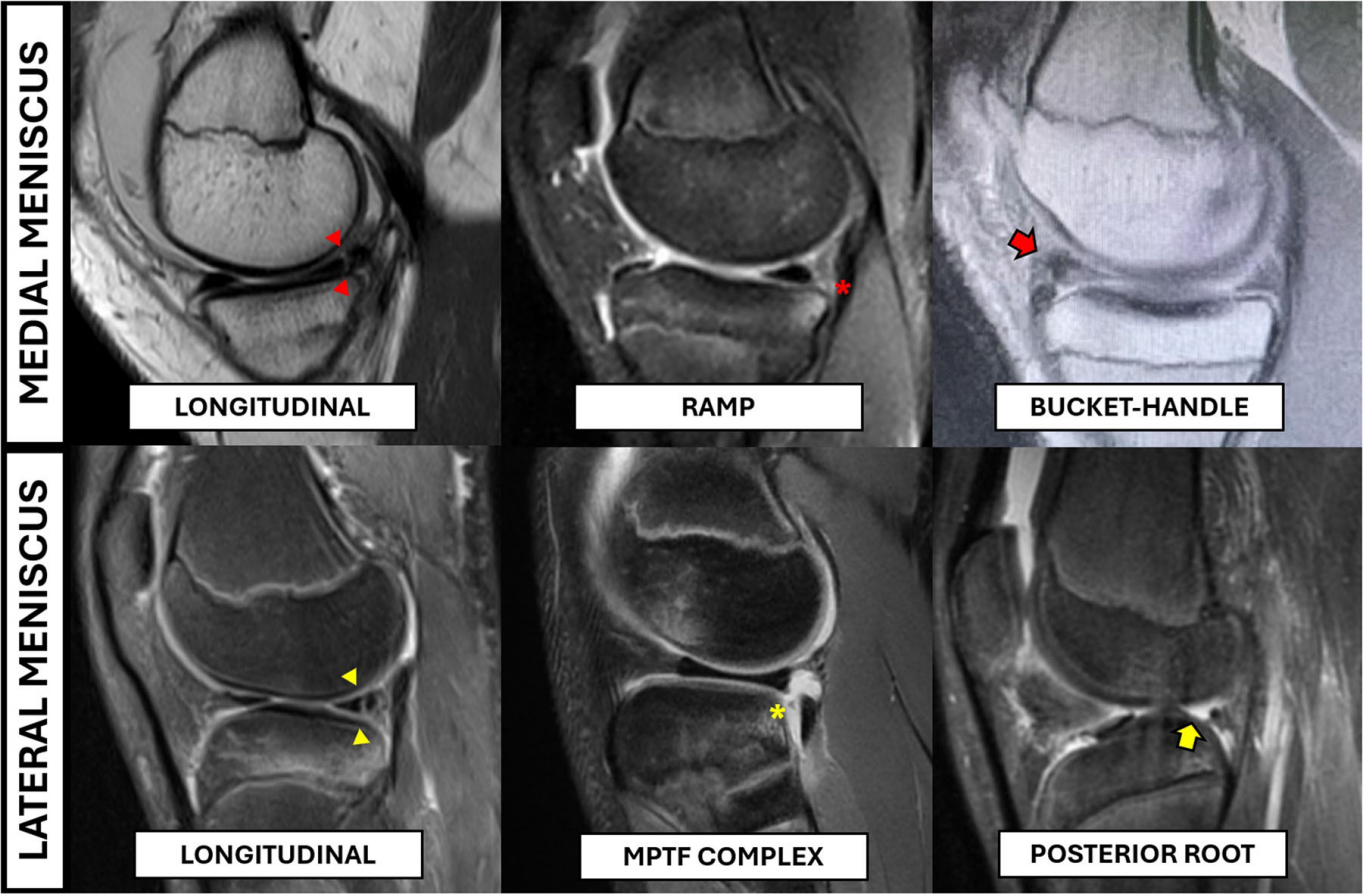
The most common different patterns of medial and lateral meniscus tears in skeletally immature patients.

The same authors also performed a prospective systematic arthroscopic assessment in 62 consecutive skeletally immature patients with an average age of 14.8 years undergoing ACL reconstruction. A total of 35% had medial ramp tears and 21% had lateral root tears (Figure 7); the incidence of ramp was even higher in patients closer to skeletal maturity (Table 3), and this lesion could represent the beginning of future bucket handle tears and other more complex tear patterns [41]. In fact, Guenther et al. [45] demonstrated an increased likelihood of having medial meniscus bucket handle tears 1 year after ACL injury.

**Table 3:** Incidence of Meniscal tears in skeletally immature patients based on the proximity to skeletal maturity.

Incidence of meniscal tears according to remining growth [40]		
	YOUNGER patients (*n* = 17)	OLDER patients (*n* = 40)
	>1 year remaining growth (%)	≤1 year remaining growth (%)
Medial or lateral meniscus	**100**	**77**
Medial meniscus	**18**	**52**
Longitudinal	0	10
Ramp	18	42
Bucket handle	0	0
Lateral meniscus	**88**	**55**
Longitudinal	29	20
Bucket handle	0	5
Posterior root	12	25
Menisco‐Tibia‐Popliteo‐Fibular complex	47	5

**Note**: Bold values indicate the total number of tears in "Medial or Lateral Meniscus", "Medial Meniscus" or "Lateral Meniscus".

**Figure 8:**
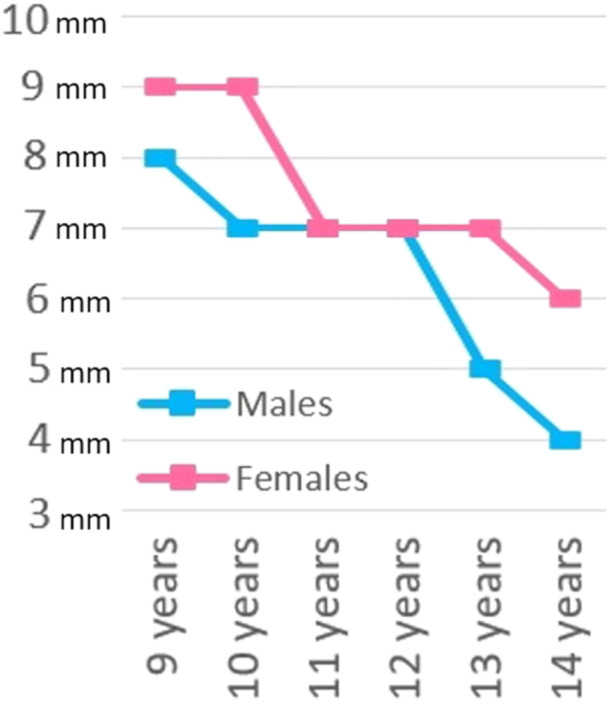
Trend of physiological knee laxity according to the skeletal age in males (blue line) and females (pink line).

For this reason, a high threshold of suspicion for meniscal injuries should be used, especially older kids. In the author's experience, patients approaching maturity should be treated more aggressively with ACL reconstruction, due to the high rate of meniscal injures, the increasing intensity of sport participation, the higher BMI and the progressive reduction of inherent knee laxity [9, 80] (Figure 8) that could reduce the tolerance of knee instability, leading to knee decompensation.

However, if a decision for a longer‐term nonoperative treatment is chosen, a systematic follow‐up with annual MRI's to evaluate the meniscal status as well as any signs of knee decompensation such as anterior tibial translation or buckling of the PCL, other than secondary meniscal tears.

### Practical algorithm for management of acute ACL

**Figure 9:**
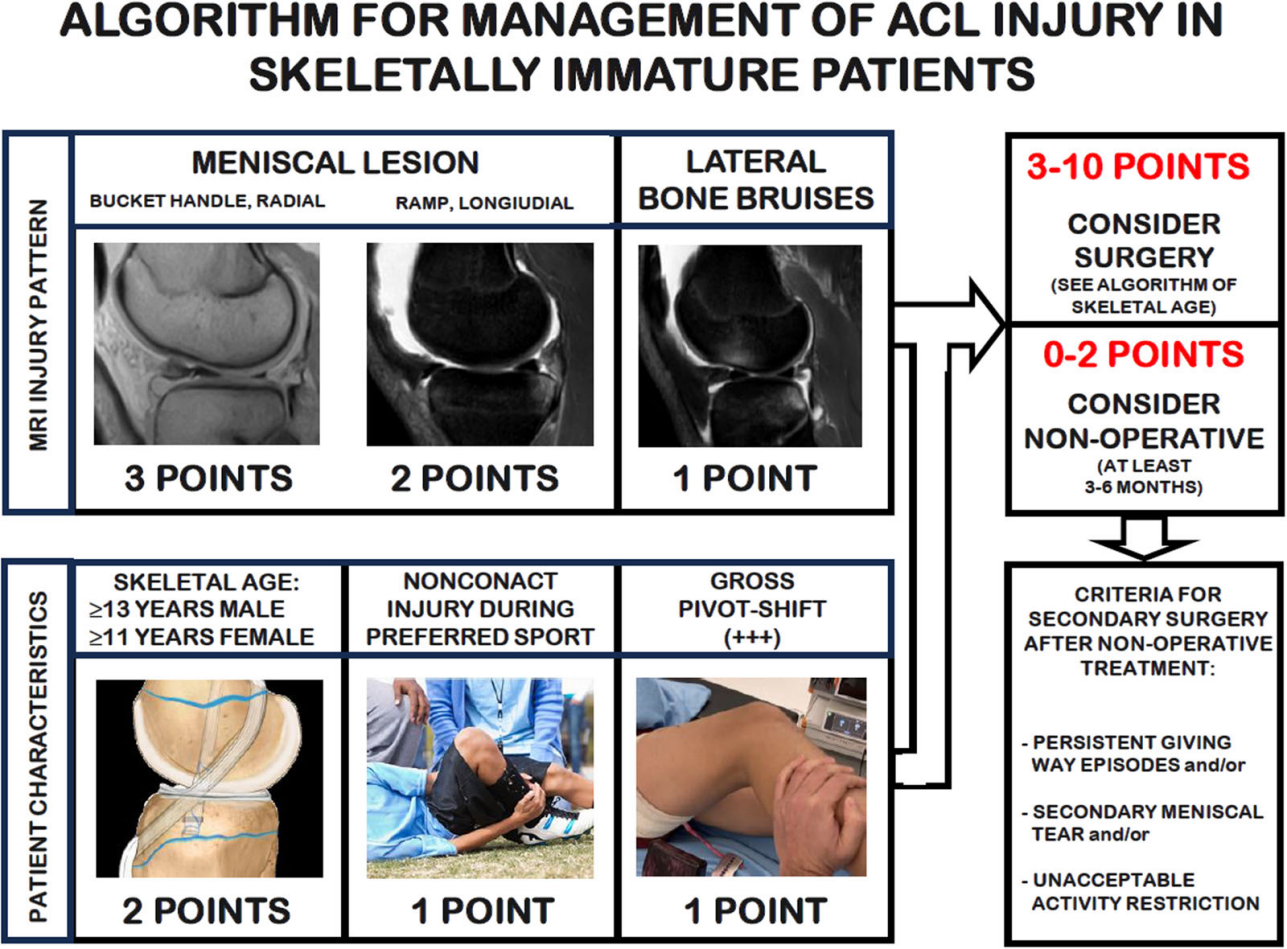
Visual representation of the BABY knee Algorithm for the management of acute anterior cruciate ligament (ACL) rupture in skeletally immature patients.

To help the clinician in the decision whether to choose surgical or conservative treatment after acute ACL injury in skeletally immature athletes, Grassi et al. developed and validated a decisional algorithm [40]. The BABY‐Knee algorithm consists in a 10‐point scoring system based on six items involving MRI evaluation and patients' characteristics such as bone age, meniscal tear, rotatory laxity, bone bruises and type of trauma (Figure 9). Based on the final score, nonsurgical management (0–2 points) or ACL reconstruction (3–10 points) is suggested. When the conservative treatment is chosen, it should be pursued for at least 3–6 months, carefully monitoring the patient in order to identify instability episodes or further subtle traumas. A new MRI is suggested after 3–6 months, to identify possible secondary meniscal lesions and re‐assess skeletal age. The score should be calculated again considering possible changes in MRI, skeletal age or laxity, and thus the management should be reassessed accordingly. Other indications for surgery after conservative treatment are (1) persistent giving‐way episodes during rehabilitation, (2) the presence of a secondary meniscal tear, (3) the unacceptable activity restriction to avoid instability episodes.

The BABY‐knee algorithm has been validated on 75 skeletally immature patients with ACL injury, with an average age of 13.9 ± 2.2 years; of the 20 patients that underwent conservative treatment, 60% failed. When they were evaluated according to the score, the BABY‐Knee algorithm was able to identify 91.7% of patients that failed conservative treatment and 87.5% of the patients that instead did not require ACL reconstruction. Regarding the other group of 55 patients that were treated surgically, all were scored in the category where ACL reconstruction was indicated, confirming the reliability of the score [40].

## SURGICAL TECHNIQUES FOR ACL RECONSTRUCTION IN SKELETALLY IMMATURE ATHLETES

**Figure 10:**
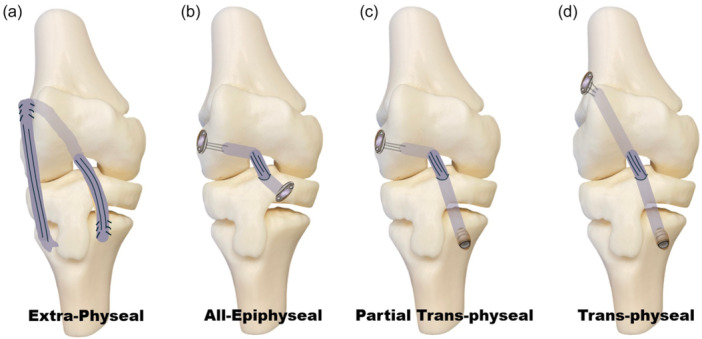
Extra‐physeal (a), all‐epiphyseal (b), partial transphyseal (c) and transphyseal (d) techniques for anterior cruciate ligament reconstruction in skeletally immature patients.

When conservative treatment fails or is not indicated, ACL reconstruction in skeletally immature patients presents several unique technical challenges, due to the need to preserve physeal integrity while ensuring functional stability. Several techniques have been developed to address these concerns, with surgical choice guided primarily by the patient's skeletal maturity and growth potential. These techniques are broadly categorised into ‘physeal‐sparing’ (extraphyseal and all‐epiphyseal) and ‘physeal‐respecting’ (partial transphyseal and transphyseal) methods (Figure 10). Moreover, lateral extra‐articular procedures (LEAP) to combine with ACL reconstructions gained interest in the last decade.

### Extraphyseal ACL reconstruction (Figure 10a)

The extraphyseal ACL reconstruction, often referred to as ‘modified Machintosh’ or ‘over‐the‐top’ technique, was popularised by Kocher and Micheli [55] for use in prepubescent children with open physes and significant growth remaining. This technique avoids the creation of any osseous tunnels, thus minimising the risk of growth disturbance. A 15‐cm strip of iliotibial band (ITB) is harvested with its distal attachment left intact at Gerdy's tubercle. The graft is then routed over the lateral femoral condyle (the ‘over‐the‐top’ position), passed intra‐articularly through the notch and under the intermeniscal ligament, and finally sutured to the anterior tibial periosteum. This technique combines intra‐articular reconstruction with an extra‐articular tenodesis and leverages the existing periosteal tension for graft fixation [55]. In a long‐term study of 137 skeletally immature patients, Kocher et al. reported excellent functional outcomes with a 6.6% graft failure rate and no growth disturbances [55]. Despite being nonanatomic, biomechanical evaluations have shown restoration of near‐normal knee kinematics, affirming its utility in the youngest cohort of patients [52].

**Figure 11:**
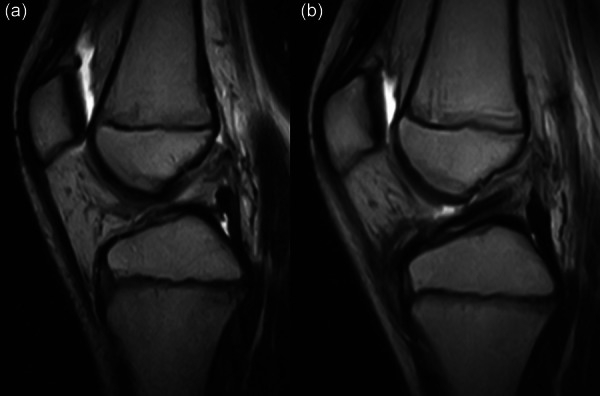
Example of a 11‐year old ‘prepubescent’ patients with anterior cruciate ligament (ACL) injury (a) and 3 months after ‘extra‐physeal’ over‐the‐top ACL reconstruction (b).

Alternatively, an ‘extra‐physeal’ modification of the original Marcacci‐Zaffagnini Over‐The‐Top and lateral tenodesis described by Grassi et al. [36] could be used to treat skeletally immature patients. This technique includes an intra‐ and extra‐articular reconstruction using a two‐strand graft of gracilis (1 strand) and semitendinosus (1 strand) which are maintained attached to the tibial insertion. Femoral and tibial fixation of the graft is performed with periosteal sutures prepubescent patients (Figure 11), metal staples in older kids [36] or alternatively soft tissue anchors as described by Monaco et al. [68].

### All‐epiphyseal (AE) ACL reconstruction (Figure 10b)

**Figure 12:**
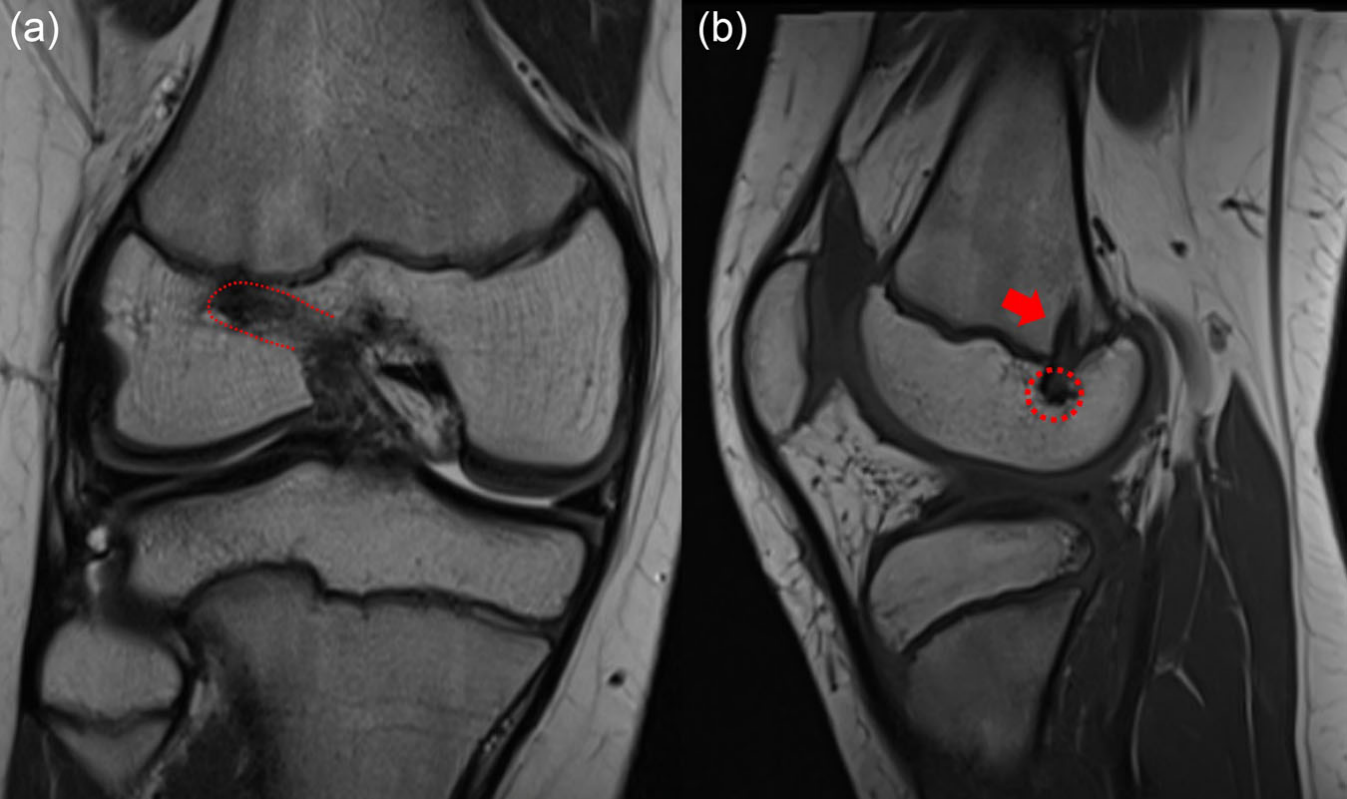
A 12.5‐year‐old male footballers after all‐epiphyseal anterior cruciate ligament (ACL) reconstruction. Despite the femoral tunnel (red dotted line) did not cross the physis (a), an asymptomatic bone bridge in the posterolateral femur (red arrow was present (b).

AE reconstruction is another physeal‐sparing technique that involves drilling both the femoral and tibial tunnels entirely within the respective epiphyses [4]. It is typically reserved for younger children with more than 2 years of growth remaining but whose epiphyseal size allows for safe tunnel placement. Originally described by Anderson [3], and further refined by Lawrence et al. [59] and McCarthy et al. [61], this technique utilises a suspensory fixation on the femoral side and metaphyseal fixation on the tibial side using soft tissue grafts, usually quadrupled hamstrings and more recently also soft‐tissue quadriceps tendon [25]. Fluoroscopic guidance is used to ensure tunnel placement avoids the physes, even if inadvertent femoral physis violation has been reported to occur in up to 43% of cases [2] (Figure 12).

Early results from Anderson's cohort demonstrated safe and effective results in patients as young as 11.1 years and with tunnel diameters ranging from 6 to 8 mm [3]. In a systematic review, comparable failure rates between all‐epiphyseal and extra‐physeal ITB techniques (9% vs. 7.2%, respectively) were reported [87], with a complication rate of 16.5% [26].

**Figure 13:**
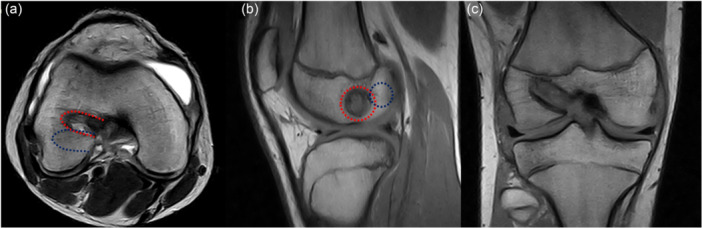
Failure of all‐epiphyseal anterior cruciate ligament (ACL) reconstruction 10 months after surgery in a 14.8‐year‐old male footballer due to tunnel malplacement (red dotted line) on axial (a), sagittal (b) and coronal view (c). The tunnel was too anterior respect of the ideal (blue dotted line) placement.

While the AE technique offers the advantage of a more anatomic graft placement compared to over‐the‐top methods, some studies suggest lower rotational control compared to ITB reconstructions [52], raising consideration in high‐demand athletes (Figure 13).

### Partial transphyseal ACL reconstruction (Figure 10c)

Partial transphyseal, or ‘hybrid’ ACL reconstruction, merges a physeal‐sparing femoral tunnel (either all‐epiphyseal or over‐the‐top) with a transphyseal tibial tunnel [21]. It is particularly suited for patients in early puberty, where the femoral physis still contributes significantly to limb length, and thus warrants preservation, while the tibial physis can be safely crossed due to earlier closure and lesser contribution to growth. It represents a technically easier procedure, which, however, could entail potential risks or complications. In fact, Chambers et al. further highlighted a growth disturbance rate of 16.7%, with a higher risk observed in patients with over five years of growth remaining. Also, Carrozzo et al. [20] reported a similar rate of 15.8% in slightly older patients, together with a 4‐year failure rate of 12.5%.

While the technique provides a compromise between anatomical reconstruction and physeal safety, it requires careful patient selection and should be avoided in the youngest patients.

### Transphyseal ACL reconstruction (Figure 10d)

**Figure 14:**
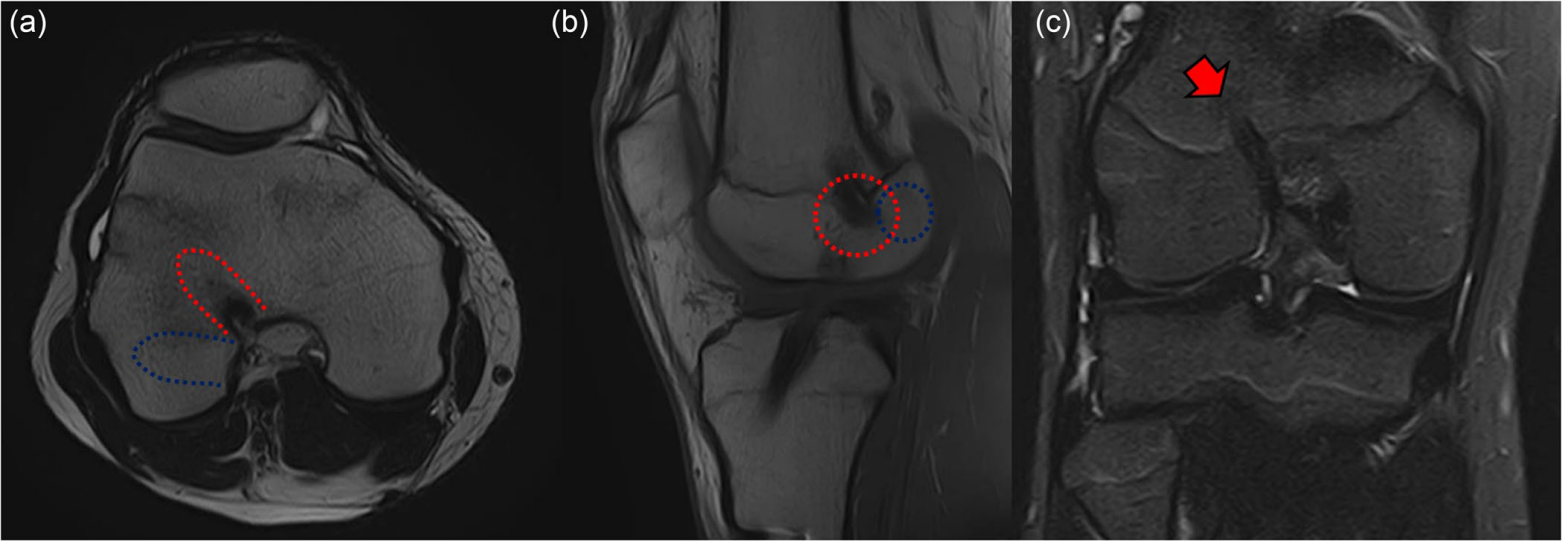
Failure of trans‐physeal anterior cruciate ligament (ACL) reconstruction 13 months after surgery in a 14.5‐year‐old male footballer due to tunnel malplacement (red dotted line and red arrow) on axial (a), sagittal (b) and coronal view (c). The tunnel was too vertical respect of the ideal (blue dotted line) placement.

Transphyseal ACL reconstruction involves creating tunnels that cross both the femoral and tibial physes, like normal standard ACL reconstruction. However, to minimise growth disturbance, it should adhere to several principles which were defined through experimental and animal works [81]. These principles involve the creation of tunnels which diameters should not exceed 8 mm to avoid >7% physeal volume violation, orientation of tunnels as vertically as possible to minimise physeal surface area disruption, and graft fixation that should avoid crossing the physis with bone plugs or metallic implants. In order to respect these rules, drilling the femoral tunnel through the anteromedial portal could increase the risk of horizontal tunnel and thus physeal damage. On the other side, vertical tunnels drilled with the transtibial method would decrease the risk of physeal damage but could increase the risk of nonanatomical tunnel placement and thus causing residual rotatory instability (Figure 14).

With transphyseal ACL reconstruction, Kocher et al. [58] showed excellent results with a 3% failure rate and no growth disturbances in 61 patients with an average age of 14.7 years. However, Bartek et al. reported a failure rate of 26% in patients with an average age of 13.6 years [8] and Fauno et al. [31] noted minor growth disturbances in 24% younger patients with an average age of 11.9 years.

For these reasons, transphyseal reconstruction remains a versatile technique for patients nearing skeletal maturity with less than 2 years of growth remaining, provided that appropriate precautions are taken in tunnel placement, diameter and fixation method.

### LEAP

As reinjury rates remain high in skeletally immature patients, particularly in those with hyperlaxity or highly active, anterolateral reinforcement has emerged as an adjunct to primary ACL reconstruction. In fact, the combination of a lateral extra‐articular tenodesis (LET) with ACL reconstruction has been demonstrated to significantly reduce the risk of failure of nearly one third (from 11% to 4%) in adult population when hamstrings are used [35]. Due to these brilliant results, LEAP are now adapted and implemented in paediatric patients with appropriate skeletal considerations [19].

Among the available options, the ‘over‐the‐top’ techniques include a LET as part of the standard procedure [36]. Using a tenodesis with the ITB, Kocher et al. reported a failure rate of 6.6% in a cohort of 137 skeletally immature patients [56], while Roberti di Sarsina et al. reported no failures in 20 skeletally immature patients when using a tenodesis with the hamstrings [79]. Another popular option for lateral tenodesis in young patients is the modified Lemaire, which is performed harvesting a strip of the ITB while maintaining its distal insertion at the Gerdy tubercle. The graft is routed deep to the lateral collateral ligament (LCL) and fixed proximally to the distal femur, often posterior and proximal to the LCL insertion. In paediatric patients, fluoroscopic guidance is essential to avoid violating the distal femoral physis during femoral fixation [36, 79]. Perelli et al. reported a reduction of failure rate from 14.7% to 6.3% when a Modified Lemaire was performed with ACL reconstruction in 66 skeletally immature patients aged between 12 and 16 years [76]. Green et al. reported no failures at 2 years in 49 patients with an average age of 14.2 years when the LET was associated with quadriceps tendon ACL reconstruction [43].

Other Lateral Extra Articular Procedures have been utilised as well. The Cocker‐Arnold [69] technique utilise and ITB strip, which is looped around the LCL and sutured on itself, thus not requiring fluoroscopy for osseous fixation. Monaco et al. [69] reported a reduction of failure rate from 15% to 0% using the latter approach. Finally, the anterolateral ligament (ALL) Reconstruction with gracilis tendon has been employed as well in skeletally immature patients by Foissey et al. [32], which reported good results and similar failure rate compared to a LET with the ITB (0% vs. 5%).

## PRINCIPLES OF REHABILITATION AND RETURN TO SPORT AFTER ACL RECONSTRUCTION

When considering the rehabilitation principles to be implemented in the optimisation of the recovery process of skeletally immature athletes affected by ACL injury, it is paramount to start from the framework. As for traditional rehabilitation, the recovery process should be structured in stages [84], ideally including Early‐ [17], Mid‐ [14] and Late‐stage [13], followed by a return to sport continuum process [13]. Despite the abovementioned similarities with traditional rehabilitation for skeletally mature athletes, children should not be considered ‘small adults’ [6], and rehabilitation must be tailored to the specific of the individuals. The whole recovery process after an ACL injury can be lengthy and challenging for children: to counteract boredom and enhance compliance, setting short‐term goals that enhance engagement is vital [60]. Paediatric rehabilitation should be performed in a multidisciplinary team in collaboration with the child's parents or guardians [6, 60] to support motivation and optimal exercise execution. Furthermore, exercise intervention should be variable and designed with a playful emphasis to avoid monotony [6]. Frequent re‐assessment of the knee joint status is fundamental in this fast‐paced developing population, as sudden changes could be overlooked [6]. Due to the intricacies of this process, high‐quality supervised rehabilitation by qualified and specialised clinicians is advocated for skeletally immature athletes.

### Early stage

When delving into the specifics of the recovery process, as for traditional rehabilitation for adults, the early‐stage rehabilitation main objectives include managing pain, reducing swelling, restoring range of motion (ROM), muscle re‐activation and functional recovery of the activities of daily living [17]. Despite the effectiveness of a bracing protocol post‐ACL injury or reconstruction in paediatric patients remains currently unknown, it is common practice to prescribe a protective brace in the early stages and at the return to unrestricted activity. Aside from the physical support in preventing knee varus‐valgus and hyperextension, the use of a brace may increase a child's awareness of the injury and play as a social‐protective message to other children during activities of daily living or sports [6], but on the other side brace can also impair sport gestures where dexterity is required. Typical criteria implemented to progress from this stage include minimal pain, absence to trace of effusion, full knee extension to ≥120° of flexion, optimal gait pattern, ability to achieve active knee extension and quadriceps engagement [6, 17, 60].

### Mid‐stage

In this phase, which typically commences after the resolution of the acute symptoms and continues until the achievement of criteria to progress to the late‐stage, the main objectives include recovering muscle strength, improving movement quality and restoring general fitness [14]. It is of vital importance to note that this rehabilitation stage is the most variable when compared with adults and paediatric patients of different age groups. As highlighted by the IOC [6], rehabilitation must be tailored to the patient's psychological and physiological maturity. Rehabilitation for prepubescent children who rely more on neural adaptations to drive neuromuscular improvements, should pose additional focus on movement quality restoration and less on resistance training [6, 60]. Typical criteria to progress to the next rehabilitation stage include full ROM, strength measurements (such as isokinetic dynamometer testing for quadriceps and hamstrings) with ≥80% limb symmetry index (LSI) (especially for adolescent patients, less strict for prepubescent children), ability to jog for 10 min without adverse reactions, and finally ≥80% LSI on functional performance testing (e.g., hop testing), ideally including not only distance metrics, but also movement quality objective scores [6, 14, 28, 60].

### Late‐stage

It involves patients resuming practice in their familiar environment but still in a controlled and restricted manner. The main objectives in this rehabilitation stage involve optimising neuromuscular performance, movement quality, sports technique, physical conditioning and restoring training loads [13]. Despite the lack of research on the introduction of specific late‐stage rehabilitation framework in skeletally immature populations based on the athlete's practised sport, on‐field rehabilitation (OFR) for soccer players [15, 16], showed promising results in adults [77], highlighting the potential benefits of implementation of the same concepts into skeletally immature population.

### Return to play

Finally, in the return to sport continuum, currently consisting of return to training, return to play and ultimately return to performance [13], patients gradually reintegrate into their sport in a controlled manner, to return to express the same performance as before the injury. Typical suggested criteria in this population include absence of pain and effusion, full knee ROM, no functional instability episodes, optimal strength (≥90%–95% LSI on isokinetic dynamometer assessment), nearly symmetrical function with optimal movement quality (e.g., ≥95% hop test battery), successful gradual involvement in sport‐specific training without adverse reactions, confidence and mentally readiness to RTS [6, 7, 60].

## OUTCOMES OF ACL RECONSTRUCTION IN SKELETALLY IMMATURE ATHLETES

### Clinical results

In the last two decades, multiple studies investigating the outcomes of ACL reconstruction in skeletally immature patients has been published; however, the great variability of patients features according to the age and developmental status, the substantial differences between male and female adolescents and the multiple surgical technique options leave this field still non completely explored.

Regarding the general results of ACL reconstruction in skeletal immature patients, systematic review by Migliorini et al. [65] including 1691 patients with an average age of 12.7 years demonstrated the efficacy of the procedure by reporting a significant improvement in the overall IKDC scores by a mean of 39.4 points, in Lysholm by 22.5, and in Tegner activity level by 1.8 points. Moreover, 89% of patients returned to sport and 84% at their preinjury level, after a mean of 8.3 ± 1.9 months. However, the most relevant outcome was graft re‐tear, with occurred in 9% of cases. No clear superiority of a technique over the other have been reported, even if a huge variability of failure has been reported, ranging from 0% with all‐epiphyseal soft tissue quadriceps graft plus LET [43] to 26% with transphyseal quadrupled hamstrings [8]. A recent systematic review suggested superior functional recovery with shorter time to RTS (7.7 vs. 8.6 months), and higher return to sport rate (99% vs. 93%) using the all‐epiphyseal technique compared to the transphyseal technique [66]. Despite being considered a well‐known option for prepubescent patients, the extraphyseal ITB (Kocher‐Micheli) technique has not been studied extensively. However, the few reports available show an encouraging graft failure, which is even lower compared to the all‐epiphyseal technique, though not to a statistically significant degree (7.9% vs. 3.6%) [53]. Lastly, in the panorama of surgical techniques, ACL repair has demonstrated unacceptably high failure rate in skeletally immature patients, thus discouraging its use [53].

More recently, the ‘paediatric ACL: understanding treatment options (PLUTO)’ study group in the United States set out to follow skeletally immature patients treated at 10 hospitals for ACL tears for 5–10 years (www.plutoacl.org). The 2‐year outcome data of ACL reconstruction in 742 patients with a mean age of 12.9 ± 1.9 years old were recently presented by Mininder Kocher at the 2024 ACL Study Group in Niseko, Japan (personal communication): graft rupture was seen in 7% overall, with lower rates in the extraphyseal ITB (3%) and all‐epiphyseal (3%) techniques as compared partial transphyseal (8%) and transphyseal (10%) techniques. Regarding the type of surgery, there was no clear indication on which technique to employ in a specific age category. For example, an adolescent with 2 years of growth remaining may reasonably be considered for either an All‐Epiphyseal or Transphyseal technique, while a patient with 3–4 years of growth remaining could be equally treated with either Extraphyseal or all‐epiphyseal techniques. In these young patients with relevant growth potential, one determining factor to choose between the two options could be the size of tibial bony epiphyses. In fact, in those under 10–11 years old, the epiphysis could be too small to safely place all‐epiphyseal tunnels without undue risk of physeal injury. Thus, some authors suggest the use of extraphyseal techniques in these smaller knees, even though they may prefer all‐epiphyseal techniques in slightly older patients [4].

### Growth disturbances

**Figure 15:**
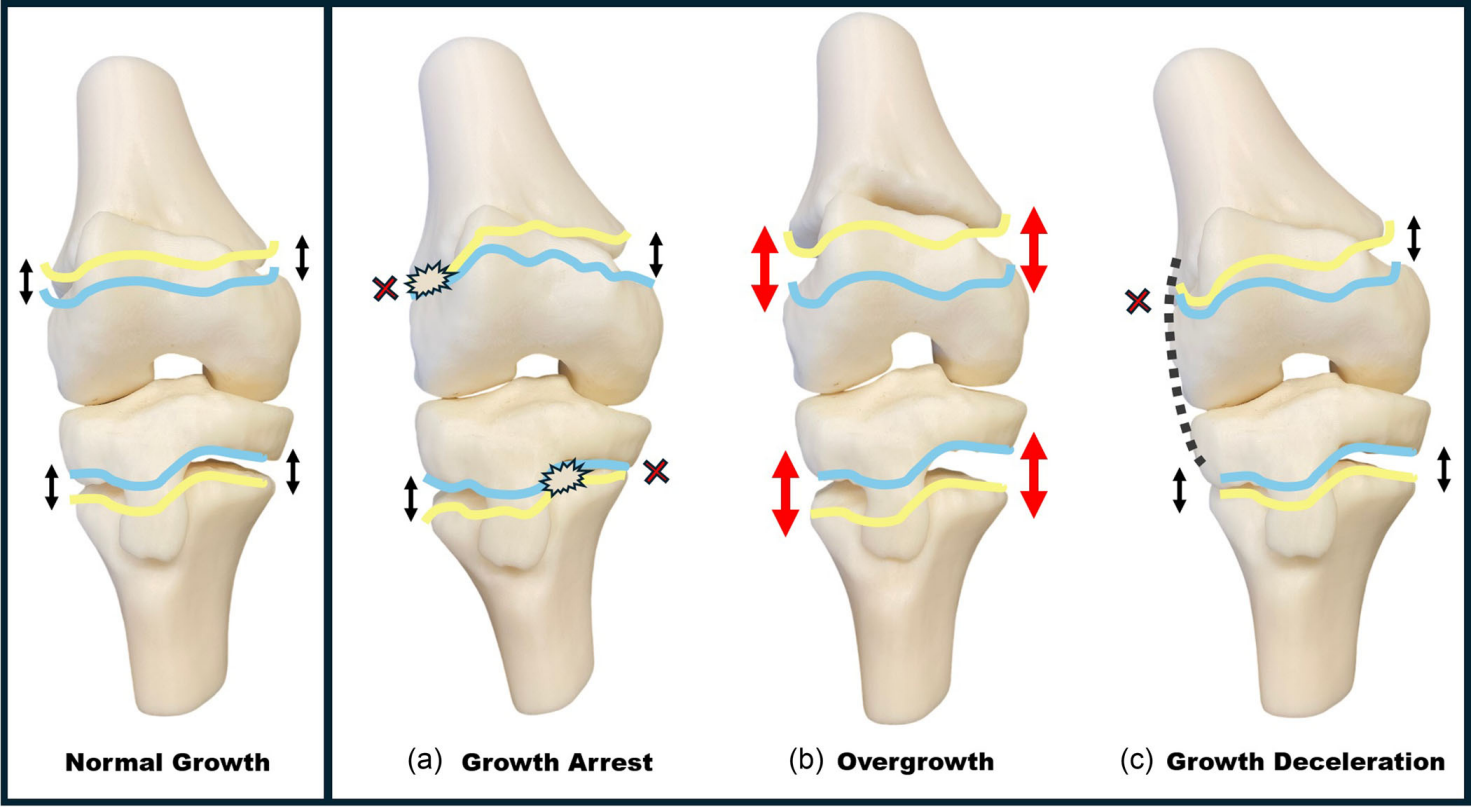
Schematic representation of the possible growth disturbances after anterior cruciate ligament (ACL) reconstruction in patients with open physes: growth arrest (a), overgrowth (b), growth deceleration (c). Physeal growth is represented by the distance between the yellow and blue lines. The black arrows represent normal growth; the big red arrows represent increased growth while the red ‘X’ represent growth arrest. Black dotted line represents lateral tenodesis.

Growth disturbances after ACL reconstruction in patients with open physes represent probably the most feared complication. Though rare, they can influence limb alignment and length and occasionally require secondary procedures. Growth disturbances after ACL reconstruction can be classified into three main categories based on their underlying mechanism and clinical consequences: Type A (growth arrest), Type B (overgrowth) and Type C (growth deceleration) [22] (Figure 15).

#### **Type A—Growth arrest (**Figure 15a **)**

This is the first and most severe type of disturbance, which occurs when the growth plate is damaged, typically by surgical trauma such as transphyseal drilling, improperly placed hardware, or a bone plug crossing the physis [81]. This trauma can cause the formation of a bone bridge, halting growth either locally or entirely; if this bridge forms centrally, it may cause symmetrical leg length discrepancies, while a peripheral location can lead to angular deformities such as genu valgum or recurvatum. The femoral side is particularly at risk if posterior structures like the Ranvier zone or perichondral ring are injured; [81] likewise, on the tibial side, damage to the tibial tuberosity apophysis may result in anterior bowing or recurvatum. Considering the late ossification of the femoral physis and the impossibility to drill a central tunnel on the femoral side, this area remains the main concern during surgical procedures, and its preservation is probably the most challenging part of ACL reconstruction.

It is interested to note that younger children tend to have a greater regenerative capacity and may spontaneously ‘break’ small bone bridges. This capacity declines with skeletal maturity, making adolescents more susceptible to permanent deformity, even though the total growth remaining is less and thus the magnitude of the possible deformity is negligible [23].

#### Type B—Overgrowth (Figure 15b)

This type of growth disturbance, also referred to as a ‘boost’, entails an increased growth of the operated knee (on femoral or tibial side). This phenomenon is more commonly observed in younger children and is believed to result from local hypervascularization following surgery, which stimulates the growth plate, causing the limb to grow slightly longer than the contralateral side [22, 81]. Overgrowth is typically symmetrical and moderate, with discrepancies often falling under 10 mm, and is rarely clinically significant [22, 81]. However, asymmetrical overgrowth can occasionally cause valgus deformities similar to those seen after tibial metaphyseal fractures.

#### Type C—Growth deceleration (Figure 15c)

This form of disturbance occurs when the growth process slows down due to mechanical inhibition. The leading theory here is the Hueter–Volkmann principle, which states that excessive compressive forces on a physis inhibit its activity [81]. Animal studies have suggested that tensions above 80 Newtons may be sufficient to slow growth [81], and possible causes could be an excessive graft tension across the growth plate, an overtight lateral extra‐articular tenodesis or the use of a synthetic graft. Clinically, the resulting leg length discrepancy is often mild but may become apparent over time and thus long‐term radiographic follow‐up become essential.

#### 1 Incidence of growth abnormalities

According to multiple meta‐analyses, a 2.1%–7.5% rate of major leg length discrepancy (>10 mm) has been reported after ACL reconstruction in skeletally immature patients, with shortening as the most common deformity overall, especially on the femoral side and in patients that underwent transphyseal ACL reconstruction [34, 87]. Differently, overgrowth was reported more frequently in patients who had undergone an all‐epiphyseal technique, with an incidence reaching 12% in prepubescents patients [54]. Regarding major angular deformities (≥5°), a rate of 1.3%–3.7% of has been reported: the most common deformities were femoral valgus (41%), tibial recurvatum (33%) and tibial varus (22%), and occurred mostly after transhyseal ACL reconstruction.

Overall, the cumulative risk of growth disturbances was higher with the all‐epipyseal technique compared either to the transphyesal technique (5.8% vs. 1.9%) [33, 34] and to the extra‐physeal technique (2.8% vs. 0%) [89] and thus the choice of the optimal surgical technique still remains crucial and based on the single‐case evaluation. It is also important to remark that over 60% of these growth abnormalities were managed conservatively, and only 1% of the total number of skeletally immature patients that underwent ACL reconstruction required corrective surgeries [33, 34]. In the case of major and progressive angular deviations, asymmetric ipsilateral epiphysiodesis may be necessary, while symmetric epiphysiodesis on the ipsilateral or contralateral knee may be performed in the case of symmetric overgrowth or deceleration, respectively [81].

Besides frank axial or length deformities, silent physeal damages have been described as well: Yoo et al. reported a 11.6% rate of focal physeal damage at MRI after transphyseal ACL reconstruction, although none of the cases experienced functional consequences [88]. Similarly, Nawabi et al. reported a 66% rate of minimal physeal violation at MRI with an all‐epiphyseal, however, without physeal compromise [72].

## PERSONAL APPROACH OF THE AUTHORS

The authors of this current concept manage ACL injury in skeletally immature patients as follows: a detailed interview to patients and parents is conducted, a thoughtful physical examination is performed, and the knee MRI is assessed. Tanner stage is not routinary assessed. Then, the patient's skeletal age is determined by assessing the tibial epiphysis and tuberosity maturation status on sagittal MRI (STEP shorthand). Hand radiographs are obtained only in doubtful cases and not on a rutinary base. The BABY‐Knee algorithm is therefore calculated considering age, meniscal lesions, bone bruises, laxity and sport.

### Score < 3 points: Conservative

The patient is submitted to conservative treatment, including at least 3 months of supervised high‐quality rehabilitation. After 3 months, the patient is assessed again with a new MRI. If no meniscal lesions are present and if the patient does not report instability episodes, the patient is assessed annually with a new MRI. If the patient has more than 3 instability episodes or locking\mechanical symptoms down the road, MRI and assessment are anticipated and surgery is proposed.

### Score 3–10 points: Surgery

**Figure 16:**
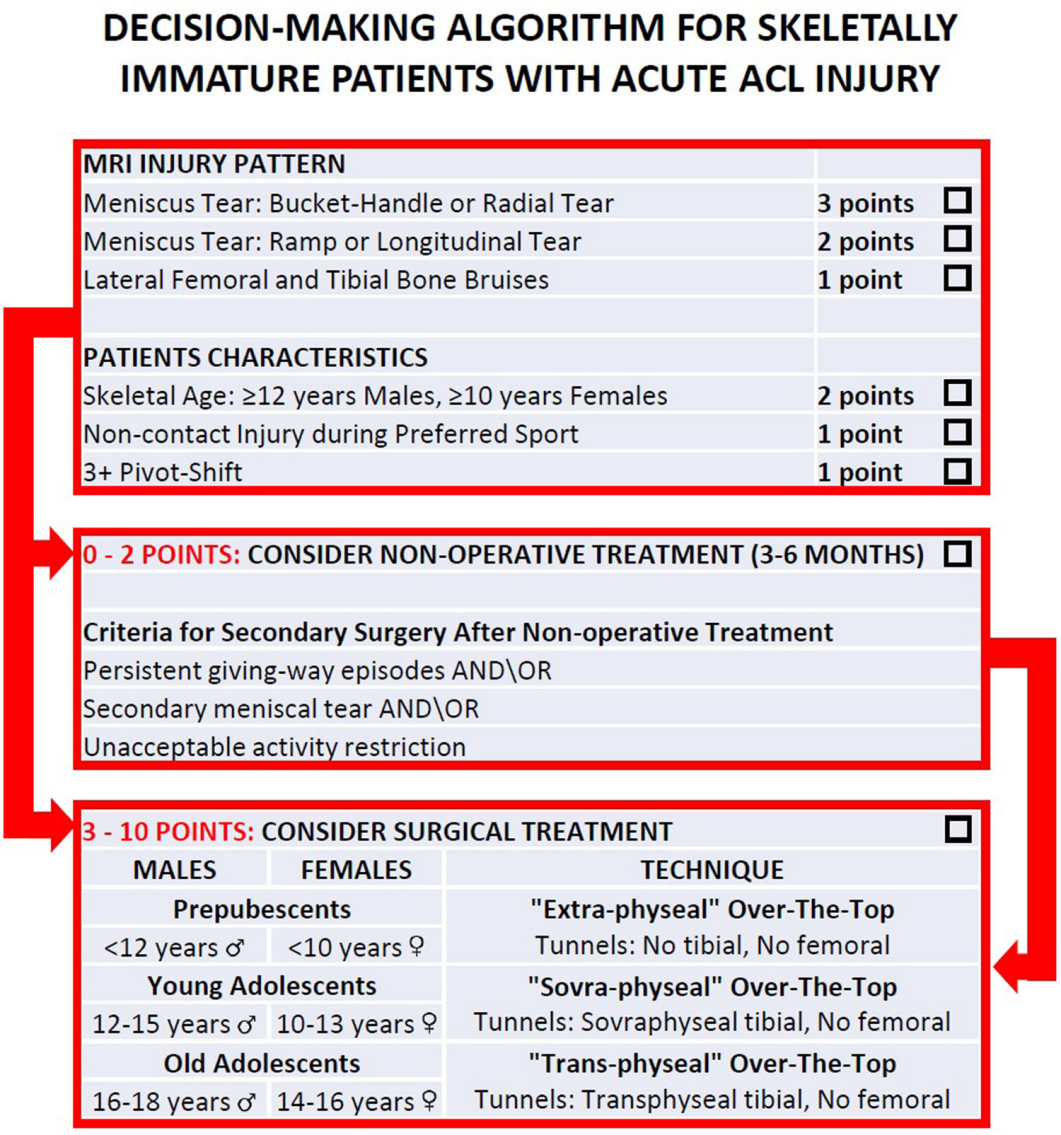
Decision‐making algorithm for the management of acute anterior cruciate ligament (ACL) injury in skeletally immature patients.

The patient is submitted to surgery after careful education of the parents regarding risks and benefits of surgery. According to skeletal age, the patient is assigned to one of the three different age groups, that determines the type of over‐the‐top technical variant (Figure 16) [36].

**Figure 17:**
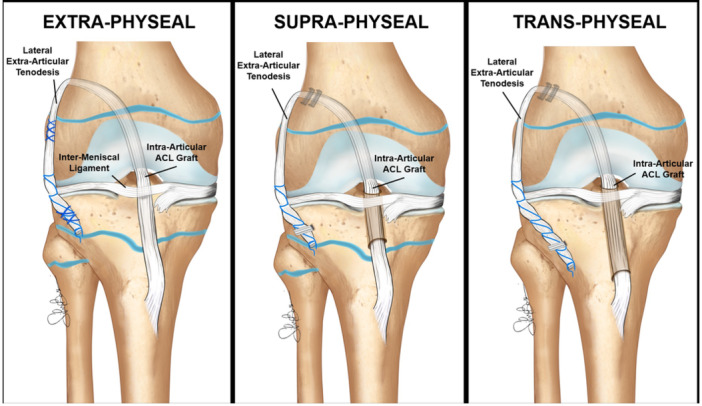
The three different ‘over‐the‐top’ options for the treatment of skeletally immature patients.

‐ *Prepubescent patients (males < 12 years and females < 10 years of skeletal age): The ‘extra‐physeal’ echnique.* The over‐the‐top and lateral tenodesis is performed in an ‘extra‐physeal’ manner, without bone tunnels (Figure 17a). This approach is similar to the Micheli technique [26], but executed in a reverse fashion, with the hamstring graft passed under the intermeniscal ligament before being secured first to the lateral femur and then to the tibia at Gerdy's tubercle utilising periosteal sutures.
‐ *Young adolescents (males 12–15 years and females 10–13 years of skeletal age): The ‘Supra‐physeal’ Technique.* The over‐the‐top and lateral tenodesis is performed in a ‘supra‐physeal’ manner (Figure 17b) by creating a tibial tunnel proximally to the tibial physis (within the proximal tibial epiphysis) and securing the graft with staples above both the femoral physis and the lateral tenodesis above the tibial physes.
‐ *Older adolescents technique (males 16–18 years and females 14–16 years of skeletal age): The ‘trans‐physeal’.* The over‐the‐top and lateral tenodesis is performed in a ‘trans‐physeal’ manner, exactly as described in the original technique utilised in adults [26], with the tibial tunnel drilled through the closed or closing tibial physis (Figure 17c).

Considering the high incidence of meniscal lesions, a thoughtful arthroscopic assessment is performed, and all efforts should be used to suture all type of lesions, usually with all‐inside devices.

Regarding the postoperative protocol, knee brace is used only in the case of bucket handle, root or longitudinal meniscus tear repair, or in prepubescent patients where compliance to full extension could be problematic. Progressive weight bearing and ROM exercises are encouraged after 3 days. Stationary bike is allowed after 2 weeks. Return to running and to recreational ludic activities is allowed since the 3rd postoperative months. In the case of competitive athletes, full return to sport is subordinate to passing the threshold value at the movement analysis test (MAT). The MAT is a comprehensive movement assessment with the function of objectively quantifying the patient's movement quality across six foundational tasks resembling the demands of multidirectional sports [27, 28, 29]. A score out of 100 is then attributed to the athlete based on the displayed kinematics, with a score of ≥85 considered acceptable for return to team training.

**Figure 18:**
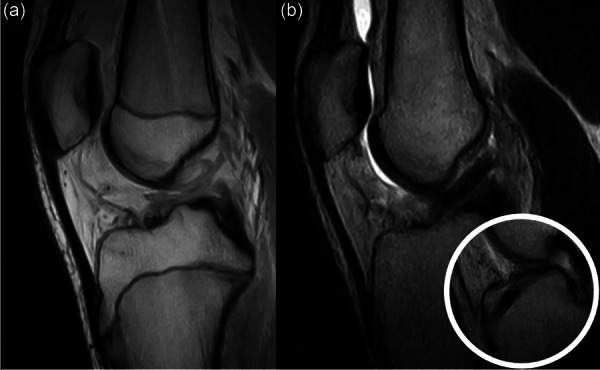
Preoperative magnetic resonance imaging (MRI) of a 14.2‐year‐old male footballer with anterior cruciate ligament (ACL) injury (a) and MRI at 2‐year follow‐up after physeal sparing over‐the‐top ACL reconstruction and Lateral Tendesis (b), showing good signal and inclination of the ACL graft.

Between April 2022 and April 2025, a total of 100 skeletally immature patients (mean age 13.7 years) with ACL injury were treated by the first author (X.X.) using the current algorithm; 12% (mean age 9.6 years ± 1.5 years) were treated conservatively, 11% (mean age 11.7 years ± 1.3 years) underwent ACL reconstruction with the ‘extra‐physeal’ technique, 41% (mean age 14.0 years ± 0.8 years) with the ‘supra‐physeal’ technique and 36% (mean age 15.7 years ± 0.9 years) with the ‘trans‐physeal’ technique. Moreover, the main results, outcomes and failure rate of ACL reconstruction with the over the top approach in skeletally immature and adolescent patients (Figure 18) are reported in Table 4 [36, 37, 38, 39, 40, 41, 42, 79].

**Table 4:** Main findings of the studies performed by the authors using the described approach.

Summary of findings from the studies of the authors using over‐the‐top approach in skeletally immature patients and adolescents						
Authors	Journal	Year	Patient n°	Mean age (years)	Follow‐up (years)	Main findings
Roberti Di Sarsina et al. [79]	*KSSTA*	2019	20	12.3	4.5	Failure rate: 0%
						Patient‐reported outcome measures (PROMs): 95% good/excellent
						Growth disturbances: 0% major, 15% minor (2 varus, 1 overgrowth)
Grassi et al. [37]	*Sports Health*	2022	151	14.8	6.0	Failure rate: 6%
						Contralateral anterior cruciate ligament (ACL): 12%
						Overall reoperation rate: 21%
						Meniscus repairability: 92% if surgery within 3 months, 46% if surgery after 12 months
						Pain during activity: 26% if intact lateral meniscus, 56% in patients with lateral meniscectomy
Grassi et al. [39]	*OJSM*	2025	43	13.3	11.0	Failure rate: 10%
						Contralateral ACL: 19%
						Overall reoperation rate: 26% (Meniscus surgery 9%, Staple removal 15%)
						PROMs: 84% good/excellent

## CONCLUSIONS

This current concept provides a deep and detailed theoretical background of ACL injury and management in patients with open physis. Moreover, a practical and pragmatic algorithm of treatment is provided based on authors’ experience and research. To optimise management and reduce the risks of either conservative or surgical treatment, a deep knowledge of the specific concepts related to paediatric patients is required.

## AUTHOR CONTRIBUTIONS

All authors contributed to the study conception and design. The first draft of the manuscript was written by Alberto Grassi and all authors commented on previous versions of the manuscript. All authors read and approved the final manuscript. We thanks Silvia Bassini for the artworks and Alessandro Compagnin for the contribution in manuscript preparation.

## CONFLICT OF INTEREST STATEMENT

Alberto Grassi and Stefano Zaffagnini are consultants from Smith & Nephew and DePuy. The remaining authors declare no conflict of interest.

## ETHICS STATEMENT

The authors have nothing to report.

## Figures and Tables

**Figure A1:**
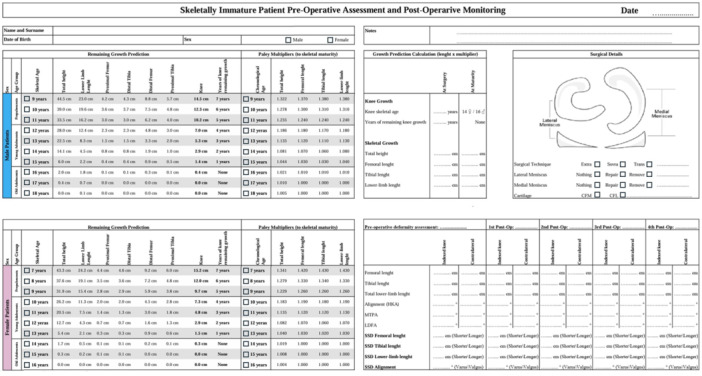
Assessment form for skeletally immature patients undergoing anterior cruciate ligament reconstruction.

## Data Availability

The authors have nothing to report.

## 1

See Figure A1.
